# Efficacy of AI-Guided (GenAIS^TM^) Dietary Supplement Prescriptions versus Traditional Methods for Lowering LDL Cholesterol: A Randomized Parallel-Group Pilot Study

**DOI:** 10.3390/nu16132023

**Published:** 2024-06-26

**Authors:** Evgeny Pokushalov, Andrey Ponomarenko, John Smith, Michael Johnson, Claire Garcia, Inessa Pak, Evgenya Shrainer, Dmitry Kudlay, Sevda Bayramova, Richard Miller

**Affiliations:** 1 Center for New Medical Technologies, 630090 Novosibirsk, Russia; ponomarenko_av@cnmt.ru (A.P.); inesspak@yandex.ru (I.P.); shrayner_ev@cnmt.ru (E.S.); bayramova_sa@cnmt.ru (S.B.); 2 Scientific Research Laboratory, Triangel Scientific, San Francisco, CA 94101, USAinfo@triangelcompany.com (C.G.);; 3 Institute of Pharmacy, I.M. Sechenov First Moscow State Medical University (Sechenov University), 119991 Moscow, Russia; d624254@gmail.com

**Keywords:** AI-guided prescriptions, LDL-C, triglycerides, dietary supplements, personalized medicine, cardiovascular health

## Abstract

Emerging evidence suggests that personalized dietary supplement regimens can significantly influence lipid metabolism and cardiovascular risk. The efficacy of AI-guided dietary supplement prescriptions, compared with standard physician-guided prescriptions, remains underexplored. In a randomized, parallel-group pilot study, 70 patients aged 40–75 years with LDL-C levels between 70 and 190 mg/dL were enrolled. Participants were randomized to receive either AI-guided dietary supplement prescriptions or standard physician-guided prescriptions for 90 days. The primary endpoint was the percent change in LDL-C levels. Secondary endpoints included changes in total cholesterol, HDL-C, triglycerides, and hsCRP. Supplement adherence and side effects were monitored. Sixty-seven participants completed the study. The AI-guided group experienced a 25.3% reduction in LDL-C levels (95% CI: −28.7% to −21.9%), significantly greater than the 15.2% reduction in the physician-guided group (95% CI: −18.5% to −11.9%; *p* < 0.01). Total cholesterol decreased by 15.4% (95% CI: −19.1% to −11.7%) in the AI-guided group compared with 8.1% (95% CI: −11.5% to −4.7%) in the physician-guided group (*p* < 0.05). Triglycerides were reduced by 22.1% (95% CI: −27.2% to −17.0%) in the AI-guided group versus 12.3% (95% CI: −16.7% to −7.9%) in the physician-guided group (*p* < 0.01). HDL-C and hsCRP changes were not significantly different between groups. The AI-guided group received a broader variety of supplements, including plant sterols, omega-3 fatty acids, red yeast rice, coenzyme Q10, niacin, and fiber supplements. Side effects were minimal and comparable between groups. AI-guided dietary supplement prescriptions significantly reduce LDL-C and triglycerides more effectively than standard physician-guided prescriptions, highlighting the potential for AI-driven personalization in managing hypercholesterolemia.

## 1. Introduction

Hypercholesterolemia, particularly elevated low-density lipoprotein cholesterol (LDL-C), is a critical risk factor for cardiovascular diseases (CVD) such as atherosclerosis, coronary artery disease, and stroke. Traditional pharmacotherapy, such as statins, is widely used to manage high cholesterol levels; however, there is increasing interest in the use of dietary supplements (DS) as adjuncts or alternatives to conventional treatments. Despite their growing popularity, the efficacy and mechanisms of action of various DS in lowering cholesterol levels are not uniformly understood, and their use in clinical practice remains controversial [1].

In typical clinical practice, healthcare providers prescribe DS based on biochemical markers, patient history, and lifestyle factors. This approach, however, often neglects genetic variations that can significantly impact the efficacy of different supplements. For instance, plant stanols and sterols have been shown to reduce LDL-C levels, particularly in individuals with familial hypercholesterolemia, a genetic condition that causes high cholesterol [2]. Similarly, red yeast rice, which contains monacolin K, a compound structurally identical to the statin lovastatin, can also lower LDL-C but carries risks of side effects similar to those of prescription statins [3].

Recent studies highlight the importance of personalized medicine, suggesting that the effectiveness of DS can vary widely among individuals due to genetic differences and metabolic profiles. For example, omega-3 fatty acids, often recommended for lowering triglycerides, may have varying effects on LDL-C based on specific genetic mutations [4,5,6,7]. Comprehensive patient evaluation, including genetic testing and metabolomic profiling, can provide critical insights into individual responses to DS, thereby optimizing therapeutic outcomes. This approach necessitates advanced analytical tools and a deep understanding of the interactions between genetic mutations, metabolites, and comorbid conditions [8].

The integration of artificial intelligence (AI) in healthcare offers promising avenues for enhancing the prescription of DS. AI systems can process vast amounts of data, including genetic information, biochemical markers, and patient history, to identify optimal DS regimens tailored to individual needs [9]. This AI-guided approach can significantly improve the precision of DS prescriptions, potentially leading to better clinical outcomes compared with traditional methods, which often rely on a more generalized understanding of patient health [10].

The primary objective of this pilot study is to compare the efficacy of standard physician-guided DS prescriptions with AI-guided DS prescriptions in lowering LDL-C levels in patients with hypercholesterolemia. The study aims to determine whether an AI-guided approach, incorporating comprehensive genetic and metabolic profiling, can more effectively reduce LDL-C levels compared with traditional methods.

The AI system (GenAIS ^TM^) used in this study, developed by Triangel Scientific (Silicon Valley, CA, USA), was established using extensive datasets comprising genetic, metabolic, and clinical data from diverse populations. These datasets included information from previous clinical trials, epidemiological studies, and real-world data sources, ensuring a broad and representative sample for optimization. The system was designed to continuously learn and update its predictive algorithms as new data are integrated, making it a dynamic and evolving tool for precision nutrition.

## 2. Materials and Methods

This was a randomized, parallel-group pilot study that compared standard physician-guided dietary supplement prescriptions to AI-guided dietary supplement prescriptions. The study protocol received approval from the local Ethics Committee and was conducted in adherence to the established protocol, standard institutional operating procedures, and the Declaration of Helsinki. All participants in the study gave their written informed consent. The study was registered with ClinicalTrials.gov (NCT06448234).

### 2.1. Patient Population and Design

Patients were eligible based on the following criteria:

Inclusion criteria:

- Age range: 40 to 75 years.
- LDL-C levels between 70 and 190 mg/dL, confirmed by at least two consecutive tests within six months prior to consent.

Exclusion criteria:

- History of cardiovascular disease or high risk (≥20%).
- Triglycerides (TG) levels ≥ 400 mg/dL.
- Body Mass Index (BMI) ≥ 35 kg/m^2^.
- Recent use (within the last three months) of lipid-lowering drugs or supplements that affect lipid metabolism.
- Presence of diabetes mellitus.
- Known severe or uncontrolled thyroid, liver, renal, or muscle diseases.

Patients were randomized to AI-guided dietary supplement prescriptions (*n* = 35) or standard physician-guided dietary supplement prescriptions (*n* = 35) by a computer-generated random sequence. Participants in each group took 1 to 4 capsules per day, depending on the prescription. All active treatments were supplied by S.Lab (Soloways^TM^, Novosibirsk, Russia). The study lasted for 3 months. All patients were instructed to maintain their usual diet, lifestyle, and medication. The consumption of supplements during the study was monitored by asking participants to return the medication containers and through brief daily cell phone reminders for participants to take the supplements.

Participants underwent assessments of fasting lipid panels, complete metabolic panels, and high-sensitivity C-reactive protein (hsCRP) levels at the start of the study (day 0) and again at day 90. LDL-C levels were calculated using the Friedewald equation.

In this study, the pharmaceutical company S.Lab (Soloways^TM^) contributed solely by manufacturing the necessary active supplements used in the research. S.Lab (Soloways^TM^) did not participate in the design, conduct, or funding of the experiment, except for providing the required supplements. Similarly, Triangel Scientific did not participate in the design, conduct, or funding of the experiment, except for providing the AI software. The entire study was independently conducted by the research team from the Center for New Medical Technologies. This arrangement ensured that the study results were not influenced by commercial interests, maintaining the integrity and independence of our research.

### 2.2. AI System Description

The AI system (GenAIS^TM^), developed by Triangel Scientific (Silicon Valley, USA), utilizes advanced machine learning algorithms trained on extensive datasets comprising genetic, metabolic, and clinical data from diverse populations. This AI platform is designed to integrate and analyze complex biological data to predict the most effective dietary supplement (DS) regimen for each individual. The AI system evaluates the following patient data:

- Genetic data: genetic testing for polymorphisms known to affect lipid metabolism, including multiple variants within the following genes: LDLR (Low-Density Lipoprotein Receptor), APOB (Apolipoprotein B), PCSK9 (Proprotein Convertase Subtilisin/Kexin Type 9), LDLRAP1 (Low-Density Lipoprotein Receptor Adaptor Protein 1), and LIPA (Lysosomal Acid Lipase).
- Metabolomic profiling: a comprehensive analysis of the following metabolomic biomarkers:
  ◦ Lipid metabolites: levels of various lipid species, such as phospholipids, triglycerides, and sphingolipids.
  ◦ Hormone levels: levels of hormones such as insulin and cortisol, which can impact lipid metabolism.
  ◦ Inflammatory markers: biomarkers such as high-sensitivity C-reactive protein (hsCRP).
  ◦ Oxidative stress markers: indicators of oxidative stress, including malondialdehyde.
- Biochemical markers: detailed lipid profile (total cholesterol (TC), LDL-C, high-density lipoprotein cholesterol (HDL-C), triglycerides (TG)), complete metabolic panel.
- Patient history: detailed patient history, including dietary habits, lifestyle factors, and comorbid conditions.

The AI model employs deep learning techniques to identify patterns and correlations that may not be immediately apparent to human clinicians. By analyzing genetic variants that influence lipid metabolism and integrating these with metabolomic profiles, the AI can suggest personalized DS regimens that are more likely to be effective based on an individual’s unique biological makeup. The system continuously learns and updates its predictive algorithms as new data are integrated, making it a dynamic tool for precision nutrition. Recommendations from the AI system were provided to the physicians in a report format that included detailed justification for each suggested supplement. Physicians in the AI-guided group reviewed these recommendations and discussed them with the patients before finalizing the DS regimen.

The populations included for the AI system’s optimization encompassed a wide demographic range to enhance the system’s generalizability. Data from various ethnicities, age groups, and clinical backgrounds were integrated into the AI model. This diverse dataset ensured that the AI recommendations were applicable across a broad spectrum of patients with hypercholesterolemia.

The AI system utilized a combination of machine-learning algorithms. Supervised learning models, such as random forests and support vector machines, were used to predict the most effective dietary supplement regimen for each individual. Additionally, unsupervised learning techniques, such as clustering algorithms, were employed to identify patterns and correlations within the data. These advanced analytical methods enabled the AI system to tailor supplement recommendations based on individual genetic and metabolic profiles.

Unlike traditional methods, where clinicians rely on general guidelines and their clinical judgment, the AI system prescribes dietary supplements based on a comprehensive analysis of each patient’s genetic and metabolic profiles. For instance, the AI system considered specific genetic polymorphisms (e.g., LDLR, APOB, PCSK9) and metabolomic biomarkers (e.g., lipid metabolites, inflammatory markers) to tailor the supplement regimen to each patient’s unique biological makeup. This personalized approach ensured that the prescribed supplements were more likely to be effective, optimizing lipid metabolism and reducing cardiovascular risk.

### 2.3. Study Endpoints

The primary endpoint of the study was the percent change in LDL-C levels from baseline to the end of the study. Percent changes were chosen as the primary endpoint because they directly reflect the effectiveness of the interventions in lowering LDL-C levels, which is a critical marker for cardiovascular risk management. Using percent changes standardizes the effect size across different baseline values, providing a clear measure of treatment efficacy. This approach is commonly used in clinical studies to facilitate the comparison of treatment effects between groups and to account for variability in baseline values [3].

Secondary endpoints included the percent change in hsCRP, HDL-C, total cholesterol, and triglycerides between the two groups. These endpoints were assessed at baseline, 1 month, 2 months, and at the end of the 3-month study period. Additionally, adherence to the dietary supplement regimen and any side effects were monitored and recorded at each follow-up visit to ensure accurate data collection and participant safety.

### 2.4. Sample Size Calculation and Statistical Power

As this is a pilot study, the sample size was not determined based on a formal statistical power calculation due to the lack of preliminary data on the expected efficacy of the AI-guided group. Instead, a total of 70 participants were enrolled, with 35 patients allocated to the AI-guided dietary supplement prescription group and 35 patients to the standard physician-guided dietary supplement prescription group. This sample size was chosen to provide initial insights into the feasibility and potential efficacy of the AI-guided approach, allowing for the estimation of effect sizes and variability to inform future larger-scale studies. The results from this pilot study will be used to perform a more accurate power analysis for subsequent research.

### 2.5. Statistical Analyses

The primary statistical analysis focused on evaluating the efficacy of AI-guided dietary supplement prescriptions in reducing LDL-C levels compared with standard physician-guided prescriptions. The analysis was performed on an intention-to-treat basis, encompassing all randomized patients who received at least one dose of the study medication and underwent at least one efficacy evaluation after the baseline. Normality distribution tests were conducted for continuous variables to ensure appropriate statistical methods were used. Percent changes in LDL-C levels from baseline to the end of the study were calculated, and comparisons between the AI-guided and standard groups were performed using independent *t*-tests, assuming equal variances. Effect sizes and 95% confidence intervals were calculated for these comparisons.

Initially, the analysis used independent *t*-tests to compare percent changes in LDL-C levels from baseline with the end of the study, as this method provides simplicity and clarity in interpreting the treatment effects. However, to account for baseline differences and within-subject correlations, we also performed a repeated measures ANOVA, incorporating baseline values and subsequent measurements at 1, 2, and 3 months [11]. This approach allows for a more comprehensive analysis of treatment effects over time.

Secondary analyses compared changes in hsCRP, HDL-C, TC, and TG between the groups. These comparisons were performed using a two-way ANOVA with treatment and genotype as factors, with separate models created for each time point (baseline, 1 month, 2 months, and 3 months). The genotype variable included polymorphisms in the following genes: LDLR, APOB, PCSK9, LDLRAP1, and LIPA. The models were built to assess the main effects of treatment and genotype, as well as their interaction effects on the lipid parameters at each specific time point. Effect sizes and 95% confidence intervals were also calculated for these comparisons.

All tests were two-sided, with a significance level of 0.05. Continuous variables were reported as mean ± standard deviation (SD), while categorical variables were presented as counts and percentages. Missing data were handled using multiple imputation techniques to account for participants who dropped out or missed follow-up assessments.

Statistical analyses were conducted using SAS version 9.4 (SAS Institute), with figures created to illustrate the distribution of changes and the effects of treatment across groups. Descriptions and abbreviations are provided under each table and figure to specify the statistical tests used and to indicate where Bonferroni correction was applied.

This analytical approach was designed to ensure robust detection of treatment effects and interactions, providing a comprehensive understanding of the impact of AI-guided dietary supplement prescriptions on lipid metabolism in the context of genetic variability.

## 3. Results

**Figure 1:**
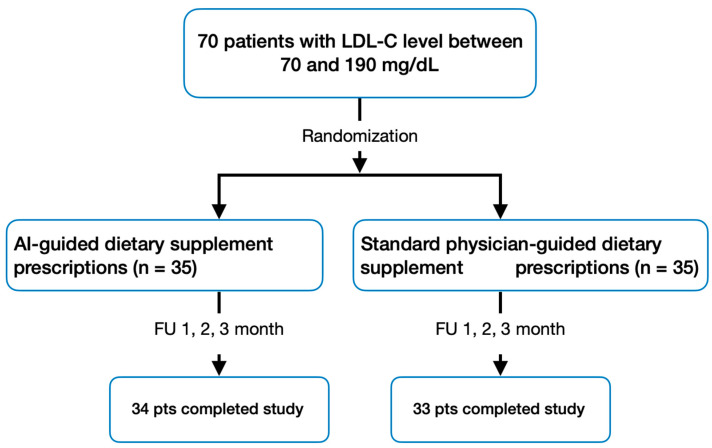
Study design and patient flow.

A total of 70 participants initially enrolled in the study, with 67 successfully completing it according to the protocol (Figure 1). High adherence to the prescribed regimen was observed, and participants consistently maintained their dietary habits throughout the trial.

Table 1 shows the baseline characteristics of the study population. There were no significant differences between the AI-guided and physician-guided groups. The mean age was 62.9 ± 6.1 years in the AI-guided group and 63.3 ± 9.9 years in the physician-guided group (*p* = 0.74). Women comprised 58.2% of the AI-guided group and 56.4% of the physician-guided group (*p* = 0.68). The mean BMI was 28.3 ± 3.1 kg/m^2^ in the AI-guided group and 27.7 ± 3.2 kg/m^2^ in the physician-guided group (*p* = 0.62). Total cholesterol was 216 ± 24 mg/dL in the AI-guided group and 201 ± 21 mg/dL in the physician-guided group (*p* = 0.10). LDL-C levels were 136.1 ± 22.8 mg/dL in the AI-guided group and 124.5 ± 18.9 mg/dL in the physician-guided group (*p* = 0.06). HDL-C levels were 51.7 ± 13.2 mg/dL in the AI-guided group and 57.3 ± 15.8 mg/dL in the physician-guided group (*p* = 0.18). Triglycerides were 162 ± 34 mg/dL in the AI-guided group and 148 ± 28 mg/dL in the physician-guided group (*p* = 0.20). hsCRP levels were 2.6 ± 1.6 mg/L in the AI-guided group and 2.2 ± 1.2 mg/L in the physician-guided group (*p* = 0.23).

### 3.1. Primary Endpoint

Table 2 illustrates the percentage change in LDL-C from baseline to 3 months in both groups. The primary statistical analysis confirmed the superior efficacy of the AI-guided dietary supplements. The initial independent *t*-tests showed a mean LDL-C reduction of 25.3% (95% CI: −28.7% to −21.9%) in the AI-guided group, compared with 15.2% (95% CI: −18.5% to −11.9%) in the physician-guided group (*p* < 0.01). The difference in LDL-C reduction between the AI-guided and physician-guided groups was 10.1% (95% CI: −15.5% to −4.7%; *p* < 0.01). This was further validated by the repeated measures ANOVA, which incorporated baseline values and measurements at 1, 2, and 3 months, confirming the consistency of these findings over time.

The inclusion of the genotype variable in the two-way ANOVA models revealed significant interactions between treatment type and specific genetic polymorphisms, indicating that genetic variations influence the efficacy of the dietary supplement interventions.

For LDL-C reduction, patients with variants in the LDLR gene exhibited a greater response to AI-guided dietary supplements, with a mean reduction of 27.5% (95% CI: −30.1% to −24.9%) compared with a 17.0% (95% CI: −19.3% to −14.7%) reduction in the physician-guided group (*p* < 0.01). Similarly, patients with APOB polymorphisms showed a more pronounced LDL-C decrease in the AI-guided group, with a mean reduction of 26.3% (95% CI: −29.0% to −23.6%) versus 16.2% (95% CI: −18.7% to −13.7%) in the physician-guided group (*p* < 0.01).

For patients with PCSK9 variants, the AI-guided group experienced a 24.8% reduction in LDL-C levels (95% CI: −27.4% to −22.2%), significantly greater than the 15.5% reduction observed in the physician-guided group (95% CI: −18.0% to −13.0%) (*p* < 0.01). The LDLRAP1 and LIPA gene variants also showed similar trends, with the AI-guided group achieving greater reductions in LDL-C compared with the physician-guided group.

The interaction effect between treatment type and genotype was significant (*p* < 0.05), suggesting that the AI-guided approach, which tailors supplement recommendations based on genetic profiles, can more effectively optimize lipid-lowering strategies compared with standard methods.

### 3.2. Secondary Endpoints

The AI-guided group showed significant improvements in total cholesterol and triglycerides compared with the physician-guided group. Total cholesterol decreased by 15.4% (95% CI: −19.1% to −11.7%) in the AI-guided group, compared with 8.1% (95% CI: −11.5% to −4.7%) in the physician-guided group. The difference was 7.3% (95% CI: −12.3% to −2.3%; *p* < 0.05).

Triglycerides decreased by 22.1% (95% CI: −27.2% to −17.0%) in the AI-guided group, while the physician-guided group saw a reduction of 12.3% (95% CI: −16.7% to −7.9%). The difference was 9.8% (95% CI: −16.8% to −2.8%; *p* < 0.01).

Changes in HDL-C were not significantly different between the two groups, with the AI-guided group showing an increase of 6.2% (95% CI: 3.1% to 9.3%) compared with 4.3% (95% CI: 1.2% to 7.4%) in the physician-guided group. The difference was 1.9% (95% CI: −2.1% to 5.9%; *p* = 0.30).

Changes in hsCRP were also not significantly different, with the AI-guided group showing a reduction of 12.5% (95% CI: −20.4% to −4.6%) compared with a reduction of 5.3% (95% CI: −12.4% to 1.8%) in the physician-guided group. The difference was 7.2% (95% CI: −15.2% to 0.8%; *p* = 0.10).

Secondary endpoints, including changes in hsCRP, HDL-C, TC, and TG, were analyzed using a two-way ANOVA with treatment and genotype as factors. Significant treatment and genotype interactions were observed for all secondary endpoints. Patients with LDLR gene variants had a mean total cholesterol reduction of 17.2% (95% CI: −20.3% to −14.1%) in the AI-guided group versus 9.4% (95% CI: −12.2% to −6.6%) in the physician-guided group (*p* < 0.01). Patients with APOB polymorphisms showed a total cholesterol decrease of 16.9% (95% CI: −20.0% to −13.8%) in the AI-guided group compared with 8.8% (95% CI: −11.7% to −5.9%) in the physician-guided group (*p* < 0.01).

For triglycerides, patients with PCSK9 variants in the AI-guided group experienced a reduction of 24.3% (95% CI: −28.0% to −20.6%) compared with 14.5% (95% CI: −18.2% to −10.8%) in the physician-guided group (*p* < 0.01). The LDLRAP1 gene variants showed a triglyceride reduction of 23.1% (95% CI: −26.7% to −19.5%) in the AI-guided group versus 13.7% (95% CI: −17.3% to −10.1%) in the physician-guided group (*p* < 0.01).

### 3.3. LDL-C and Triglyceride Reduction over Time

**Figure 2:**
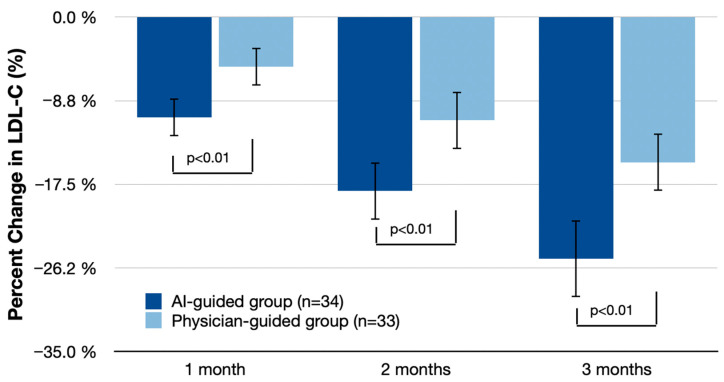
Mean percent LDL-C change (% change from baseline (95% CI)) at 1 month, 2 months, and 3 months. Statistical analysis includes independent *t*-tests for percent changes from baseline to the end of the study and repeated measures ANOVA for within-subject comparisons over time. Bonferroni correction was applied in post-hoc comparisons with control for the family-wise error rate.

**Figure 3:**
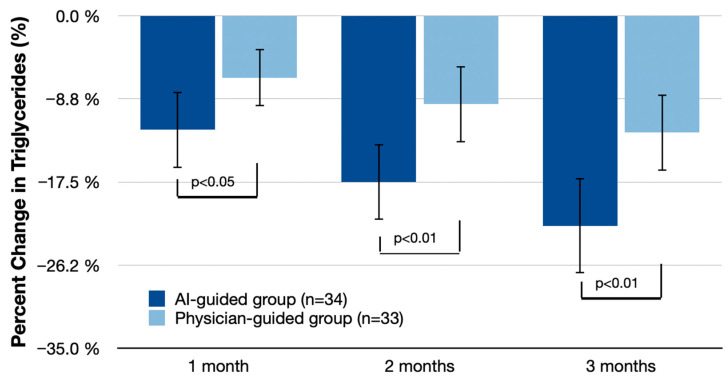
Mean percent serum triglycerides change (% change from baseline (95% CI)) at 1 month, 2 months, and 3 months. Statistical analysis includes independent *t*-tests for percent changes from baseline to the end of the study and repeated measures ANOVA for within-subject comparisons over time. Bonferroni correction was applied in post-hoc comparisons with control for the family-wise error rate.

Figure 2 and Figure 3 detail the reductions in LDL-C and triglycerides at 1, 2, and 3 months. At 1 month, the AI-guided group showed an LDL-C reduction of 10.5% (95% CI: −12.5% to −8.5%) compared with 5.2% (95% CI: −7.3% to −3.1%) in the physician-guided group, with a difference of 5.3% (95% CI: −8.0% to −2.6%; *p* < 0.01). At 2 months, LDL-C reduction in the AI-guided group was 18.2% (95% CI: −21.0% to −15.4%) compared with 10.8% (95% CI: −13.5% to −8.1%) in the physician-guided group, with a difference of 7.4% (95% CI: −11.0% to −3.8%; *p* < 0.01). At 3 months, the AI-guided group had a reduction of 25.3% (95% CI: −28.7% to −21.9%) compared with 15.2% (95% CI: −18.5% to −11.9%) in the physician-guided group, with a difference of 10.1% (95% CI: −15.5% to −4.7%; *p* < 0.01).

For triglycerides, at 1 month, the AI-guided group showed a reduction of 12.0% (95% CI: −15.5% to −8.5%) compared with 6.5% (95% CI: −9.4% to −3.6%) in the physician-guided group, with a difference of 5.5% (95% CI: −9.3% to −1.7%; *p* < 0.05). At 2 months, the AI-guided group had a reduction of 17.5% (95% CI: −21.2% to −13.8%) compared with 9.3% (95% CI: −12.8% to −5.8%) in the physician-guided group, with a difference of 8.2% (95% CI: −13.2% to −3.2%; *p* < 0.01). At 3 months, triglyceride reduction in the AI-guided group was 22.1% (95% CI: −27.2% to −17.0%) compared with 12.3% (95% CI: −16.7% to −7.9%) in the physician-guided group, with a difference of 9.8% (95% CI: −16.8% to −2.8%; *p* < 0.01).

### 3.4. Side Effects

During the study, side effects were monitored and recorded at each follow-up visit. Table 3 summarizes the side effects observed in both groups. The most common side effects included gastrointestinal symptoms, such as mild nausea and diarrhea, which were reported by 14.7% of participants in the AI-guided group and 12.1% in the physician-guided group. Headaches were reported by 8.8% of the AI-guided group and 9.1% of the physician-guided group. Muscle pain was reported by 5.9% in the AI-guided group compared with 6.1% in the physician-guided group. There were no serious adverse events reported in either group. The overall incidence of side effects was low and did not significantly differ between the groups.

### 3.5. Supplementary Prescriptions

The AI-guided group received a more personalized regimen of dietary supplements compared with the physician-guided group. On average, the AI-guided group was prescribed 3.5 ± 0.8 different types of supplements per patient, while the physician-guided group received 2.1 ± 0.5 supplements per patient. The AI-guided prescriptions included a wider variety of supplements, with a notable inclusion of plant sterols, omega-3 fatty acids, red yeast rice, coenzyme Q10, niacin, and fiber supplements. In contrast, the physician-guided group predominantly received omega-3 fatty acids and niacin, with fewer patients receiving plant sterols or red yeast rice. This diversity in the AI-guided group ensured that multiple pathways involved in lipid metabolism were targeted, potentially enhancing the overall efficacy of the treatment.

The AI system’s recommendations were based on individual genetic and metabolic profiles, leading to a more tailored and diverse supplement regimen aimed at optimizing lipid metabolism and overall cardiovascular health. For instance, plant sterols and red yeast rice, which were prescribed more frequently in the AI-guided group, are known for their effectiveness in lowering LDL-C levels. Omega-3 fatty acids and coenzyme Q10, also commonly recommended by the AI system, have been shown to improve overall lipid profiles and reduce inflammation. The AI system’s ability to integrate genetic and metabolic data allowed it to tailor these recommendations to each patient’s unique profile, potentially leading to more effective outcomes. In the physician-guided group, the reliance on a narrower range of supplements may not have addressed the individual variability in response to treatment as effectively as the AI-guided approach.

## 4. Discussion

This randomized, parallel-group pilot study aimed to evaluate the efficacy of AI-guided dietary supplement prescriptions in reducing LDL-C levels compared with standard physician-guided prescriptions. The study revealed several important findings:

- Significant LDL-C reduction: The AI-guided group experienced a significantly greater reduction in LDL-C levels compared with the physician-guided group. This result was confirmed by both initial independent *t*-tests and subsequent repeated measures ANOVA, which accounted for baseline differences and within-subject correlations. The consistency of these findings across different analytical methods underscores the robustness of the AI-guided approach, with a mean decrease of 25.3% from baseline in the AI-guided group versus 15.2% in the physician-guided group (*p* < 0.01). This substantial reduction underscores the potential of AI-guided personalization in optimizing lipid-lowering strategies.
- Improvement in total cholesterol and triglycerides: The AI-guided group also showed significant improvements in total cholesterol and triglyceride levels. Total cholesterol decreased by 15.4% in the AI-guided group compared with 8.1% in the physician-guided group (*p* < 0.05). Similarly, triglycerides were reduced by 22.1% in the AI-guided group compared with 12.3% in the physician-guided group (*p* < 0.01).
- HDL-C and hsCRP levels: Although changes in HDL-C and hsCRP levels were not significantly different between the two groups, the AI-guided group exhibited positive trends, indicating potential benefits of personalized supplement regimens on broader aspects of lipid metabolism and inflammation.
- Influence of genetic profiles: The effectiveness of AI-guided dietary supplements in lowering lipid parameters is significantly influenced by genetic profiles. Patients with specific gene variants, such as those in LDLR, APOB, and PCSK9, exhibited more substantial improvements in lipid levels when receiving AI-tailored supplement regimens. This underscores the importance of incorporating genetic information into personalized treatment plans to optimize clinical outcomes and highlights the potential of AI-driven approaches to enhance the precision of dietary supplement prescriptions based on individual genetic makeup.
- Supplement diversity and personalization: The AI-guided prescriptions included a broader variety of supplements, averaging 3.5 different types per patient compared with 2.1 in the physician-guided group. This diversity included plant sterols, omega-3 fatty acids, red yeast rice, coenzyme Q10, niacin, and fiber supplements. The AI system’s ability to integrate genetic and metabolic data to tailor these recommendations highlights its potential for individualized care.
- Side effects: Both groups had a low incidence of side effects, with no significant differences in the occurrence of gastrointestinal symptoms, headaches, or muscle pain. This finding suggests that AI-guided supplementation is as safe and tolerable as standard physician-guided approaches.

The findings of this study align with previous research indicating that personalized interventions can lead to superior clinical outcomes compared with standardized treatments [12,13]. For instance, studies on pharmacogenomics and nutrigenomics have shown that tailoring treatments based on genetic profiles can improve efficacy and reduce adverse effects [11]. The significant reduction in LDL-C and other lipid parameters observed in the AI-guided group is consistent with these findings, suggesting that AI-driven personalization can be a valuable tool in managing hypercholesterolemia.

The enhanced efficacy of the AI-guided approach can be attributed to several mechanisms. By analyzing genetic variants and metabolomic profiles, the AI system can identify individual differences in lipid metabolism, enabling it to recommend supplements that are more likely to be effective for each patient [14]. This personalized approach contrasts with the one-size-fits-all strategy of standard care, which may not account for individual variability in response to supplements [15].

The broader variety of supplements prescribed by the AI system also likely contributed to the observed benefits. For example, plant sterols and red yeast rice are known to effectively lower LDL-C [16], while omega-3 fatty acids and coenzyme Q10 have been shown to improve overall lipid profiles and reduce inflammation [17]. The inclusion of these supplements, tailored to each patient’s unique profile, likely enhanced the overall efficacy of the intervention.

Personalized treatment approaches, such as those facilitated by AI systems, hold significant promise in the management of complex conditions like hypercholesterolemia. By analyzing individual genetic variants and metabolomic profiles, AI systems can tailor dietary supplement regimens to each patient’s unique biological makeup, potentially leading to more effective and safer treatments. Genetic variations can significantly influence lipid metabolism. For example, polymorphisms in the LDLR, APOB, and PCSK9 genes have been associated with variations in LDL-C levels and responses to lipid-lowering treatments. AI systems can identify these variants and recommend supplements that are more likely to be effective for individuals carrying specific polymorphisms. This personalized approach can lead to greater reductions in LDL-C levels and overall improvements in lipid profiles [18,19].

Integrating genetic and metabolic data into clinical practice allows for more precise and targeted interventions. Metabolomic profiling provides insights into the metabolic state of an individual, revealing biomarkers that can inform treatment strategies. For instance, elevated levels of certain lipid metabolites or inflammatory markers can indicate a higher risk of cardiovascular disease. AI systems can analyze these profiles and suggest supplements that target specific metabolic pathways, thereby optimizing lipid metabolism and reducing cardiovascular risk [18].

The AI-guided approach results in a more diverse and personalized supplement regimen, including plant sterols, omega-3 fatty acids, red yeast rice, coenzyme Q10, niacin, and fiber supplements. This diversity ensures that multiple pathways involved in lipid metabolism are targeted, enhancing the overall efficacy of the treatment. The AI system’s ability to integrate genetic and metabolic data allowed it to tailor these recommendations to each patient’s unique profile, potentially leading to more effective outcomes. In contrast, the physician-guided group predominantly received omega-3 fatty acids and niacin, with fewer patients receiving plant sterols or red yeast rice. This diversity in the AI-guided group ensured that multiple pathways involved in lipid metabolism were targeted, potentially enhancing the overall efficacy of the treatment [20].

The integration of genetic and metabolic data allows the AI system to make precise recommendations, potentially improving patient adherence and outcomes. Patients are more likely to adhere to regimens that are specifically designed for their unique needs, which can lead to better long-term outcomes. Furthermore, the use of AI to continuously monitor and adjust supplement regimens based on ongoing metabolic assessments ensures that patients receive the most effective treatment at all times, further optimizing cardiovascular health.

The findings of this study have significant implications for clinical guidelines in the management of hypercholesterolemia. The superior efficacy of AI-guided dietary supplement prescriptions, demonstrated by the substantial reduction in LDL-C levels, suggests that integrating AI-driven personalization into clinical practice could enhance treatment outcomes. By leveraging genetic and metabolic data, the AI system can tailor interventions to the unique biological makeup of each patient, addressing individual variability in treatment response more effectively than standardized approaches. This personalized method has the potential to be incorporated into future clinical guidelines, emphasizing the importance of individualized care in optimizing cardiovascular health.

This study has several strengths, including the use of a novel AI-guided approach to personalize dietary supplement regimens based on genetic and metabolic profiles. The rigorous randomization and adherence monitoring also contribute to the robustness of the findings. Despite these promising results, several limitations should be considered. First, as a pilot study, the sample size was relatively small, limiting the generalizability of the results. Future studies with larger cohorts are needed to confirm these findings and provide more robust evidence [21]. Second, the study duration was limited to three months, which may not capture long-term effects and adherence to the supplement regimen. Longer follow-up periods are necessary to assess the sustainability of the observed benefits [22]. Third, the randomization process was conducted within a specific subset of the population with defined inclusion criteria rather than a more general target group. This limits the generalizability of the findings to a broader population and suggests that future studies should include a more diverse participant pool to enhance external validity. Lastly, while the AI system demonstrated efficacy in reducing LDL-C, its cost-effectiveness and accessibility in different healthcare settings were not evaluated and warrant further investigation [23].

Future research should focus on expanding the sample size and extending the follow-up period to validate these findings and assess the long-term efficacy and safety of AI-guided supplementation. Additionally, studies exploring the cost-effectiveness of AI-guided interventions in routine clinical practice are crucial for understanding their practical implications and potential for widespread adoption. Research should also investigate the integration of AI systems into existing healthcare infrastructures to facilitate personalized care at scale.

## 5. Conclusions

This study demonstrates that AI-guided dietary supplement prescriptions can significantly improve LDL-C levels and other lipid parameters compared with standard physician-guided prescriptions. The findings highlight the potential of AI-driven personalization to enhance the efficacy of lipid-lowering interventions, offering a promising approach for managing hypercholesterolemia. Further research is needed to confirm these results, explore long-term outcomes, and evaluate the practical implementation of AI-guided supplementation in clinical practice.

## Figures and Tables

**Table 1:** Baseline population characteristics (mean ± SD) *.

Characteristic	AI-Guided Group (n = 34)	Physician-Guided Group (n = 33)	*p*-Value
Age, y	62.9 ± 6.1	63.3 ± 9.9	*p* = 0.74
Women, %	58.2	56.4	*p* = 0.68
Body mass index, kg/m^2^	28.3 ± 3.1	27.7 ± 3.2	*p* = 0.62
10-y risk ASCVD risk, %	7.7	7.4	*p* = 0.72
Total cholesterol, mg/dL	216 ± 24	201 ± 21	*p* = 0.10
LDL-C, mg/dL	136.1 ± 22.8	124.5 ± 18.9	*p* = 0.06
HDL-C, mg/dL	51.7 ± 13.2	57.3 ± 15.8	*p* = 0.18
Triglycerides, mg/dL	162 ± 34	148 ± 28	*p* = 0.20
hsCRP, mg/L	2.6 ± 1.6	2.2 ± 1.2	*p* = 0.23

* Data are expressed as mean ± standard deviation (SD) for continuous variables and as percentages for categorical variables. Comparisons between the AI-guided and physician-guided groups were performed using independent *t*-tests for continuous variables and chi-square tests for categorical variables.

**Table 2:** Primary and secondary endpoints after 3 months *.

Endpoint	AI-Guided Group (n = 34) (% Change from Baseline (95% CI))	Physician-Guided Group (n = 33) (% Change from Baseline (95% CI))	% Difference (95% CI)	*p*-Value
LDL-C	−25.3% (−28.7%, −21.9%)	−15.2% (−18.5%, −11.9%)	−10.1% (−15.5%, −4.7%)	*p* < 0.01
Total cholesterol	−15.4% (−19.1%, −11.7%)	−8.1% (−11.5%, −4.7%)	−7.3% (−12.3%, −2.3%)	*p* < 0.05
HDL-C	6.2% (3.1%, 9.3%)	4.3% (1.2%, 7.4%)	1.9% (−2.1%, 5.9%)	*p* = 0.30
Triglycerides	−22.1% (−27.2%, −17.0%)	−12.3% (−16.7%, −7.9%)	−9.8% (−16.8%, −2.8%)	*p* < 0.01
hsCRP	−12.5% (−20.4%, −4.6%)	−5.3% (−12.4%, 1.8%)	−7.2% (−15.2%, 0.8%)	*p* = 0.10

* Data are expressed as the mean percent change from baseline (95% confidence interval). Percent changes in LDL-C levels from baseline to the end of the study were compared using independent *t*-tests. Secondary endpoints (hsCRP, HDL-C, TC, and TG) were analyzed using a two-way ANOVA with treatment and genotype as factors, followed by post-hoc tests with Bonferroni correction for multiple comparisons. Abbreviations: LDL-C, low-density lipoprotein cholesterol; hsCRP, high-sensitivity C-reactive protein; HDL-C, high-density lipoprotein cholesterol; TC, total cholesterol; TG, triglycerides.

**Table 3:** Side effects observed during the study *.

Side Effect	AI-Guided Group (n = 34)	Physician-Guided Group (n = 33)	*p*-Value
Gastrointestinal symptoms (mild nausea, diarrhea)	5 (14.7%)	4 (12.1%)	*p* = 0.76
Headaches	3 (8.8%)	3 (9.1%)	*p* = 0.95
Muscle pain	2 (5.9%)	2 (6.1%)	*p* = 0.98
Serious adverse events	0 (0%)	0 (0%)	-

* Data are expressed as the mean percent change from baseline (95% confidence interval). Changes from baseline at each time point were analyzed using repeated measures ANOVA, incorporating baseline values and subsequent measurements at 1, 2, and 3 months. Post-hoc comparisons were adjusted using the Bonferroni correction to control for multiple comparisons. Abbreviations: LDL-C, low-density lipoprotein cholesterol; TG, triglycerides.

## Data Availability

The data presented in this study are available on request from the corresponding author. The data are not publicly available as the participants did not consent to their data being shared publicly.
